# Implementation of continuous-variable quantum key distribution with composable and one-sided-device-independent security against coherent attacks

**DOI:** 10.1038/ncomms9795

**Published:** 2015-10-30

**Authors:** Tobias Gehring, Vitus Händchen, Jörg Duhme, Fabian Furrer, Torsten Franz, Christoph Pacher, Reinhard F. Werner, Roman Schnabel

**Affiliations:** 1 Max-Planck-Institut für Gravitationsphysik (Albert-Einstein-Institut), and Institut für Gravitationsphysik Leibniz Universität Hannover, Callinstraße 38, 30167 Hannover, Germany; 2 Department of Physics, Technical University of Denmark, Fysikvej, 2800 Kongens Lyngby, Denmark; 3 Institut für Laserphysik und Zentrum für Optische Quantentechnologien, Universität Hamburg, Luruper Chaussee 149, 22761 Hamburg, Germany; 4 Institut für Theoretische Physik, Leibniz Universität Hannover, Appelstraße 2, 30167 Hannover, Germany; 5 Department of Physics, Graduate School of Science, University of Tokyo, 7-3-1 Hongo, Bunkyo-ku, Tokyo 113-0033, Japan; 6 Institut für Fachdidaktik der Naturwissenschaften, Technische Universität Braunschweig, Bienroder Weg 82, 38106 Braunschweig, Germany; 7 AIT Austrian Institute of Technology GmbH, Digital Safety & Security Department, Optical Quantum Technology, Donau-City-Straße 1, 1200 Vienna, Austria

## Abstract

Secret communication over public channels is one of the central pillars of a modern information society. Using quantum key distribution this is achieved without relying on the hardness of mathematical problems, which might be compromised by improved algorithms or by future quantum computers. State-of-the-art quantum key distribution requires composable security against coherent attacks for a finite number of distributed quantum states as well as robustness against implementation side channels. Here we present an implementation of continuous-variable quantum key distribution satisfying these requirements. Our implementation is based on the distribution of continuous-variable Einstein–Podolsky–Rosen entangled light. It is one-sided device independent, which means the security of the generated key is independent of any memoryfree attacks on the remote detector. Since continuous-variable encoding is compatible with conventional optical communication technology, our work is a step towards practical implementations of quantum key distribution with state-of-the-art security based solely on telecom components.

## Abstract (web summary)

When quantum key distribution is composed with other secure protocols the overall security has to be guaranteed, which adds further security requirements. Here, the authors demonstrate continuous-variable quantum key distribution with composable security and one-sided-device independence.

## Graphical abstract

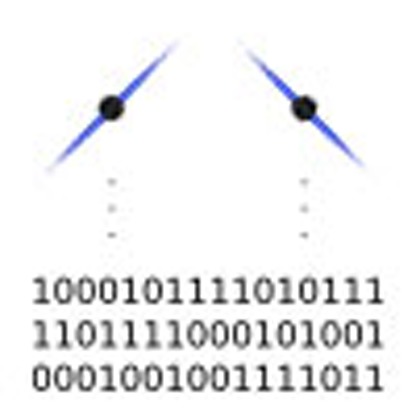

Using a quantum key distribution (QKD) system, the communicating parties employ a cryptographic protocol that cannot be broken, neither by todays nor by future technology^1^,^2^. The security of the key distributed by such a system is guaranteed on the basis of quantum theory by a mathematical proof, which has to consider the most sophisticated (quantum) attacks on the quantum channel, so-called ‘coherent attacks'. Furthermore, security has to be established in a ‘composable' fashion, which means that if the distributed key is used in another secure protocol (like one-time-pad encryption), it remains secure in the composition of the two protocols^3^,^4^. To make a security proof applicable to actual implementations, it is important to include all effects due to the finite number of distributed quantum states. In addition, the security proof has to model the source and the detectors correctly to prevent possible ‘side-channels', including those which may only be discovered in the future.

Theoretically, an elegant way to deal with imperfect sources and detectors and therefore with side channels of the implementation, is to make a proof completely device independent^5^. The found secret key rates are, however, very low so far and an implementation requires at least a detection-loophole-free Bell test, which has not been achieved in a QKD implementation so far due to inefficient detectors and photon loss in the quantum channel^5^. The idea of removing assumptions on devices can nevertheless be realized partially. For instance, measurement-device-independent QKD relies only on assumptions about the sources, located at the honest communicating parties, Alice and Bob, but not about the detectors that can be in control of the eavesdropper^6^,^7^,^8^. While in measurement-device-independent QKD the devices of Alice and Bob have to be trusted to fulfil the assumptions, it has recently been shown that QKD is even possible when the device of one of the honest parties is untrusted^9^,^10^,^11^. For discrete variables the security of this one-sided device-independent (1sDI) scheme has been analysed under the assumption on the untrusted device to be memoryless, and similar secret key rates have been obtained as in QKD implementations with trusted devices only^9^,^10^,^12^. Using continuous variables (CVs) 1sDI QKD has been recently proven secure for collective attacks and infinitely many quantum state distributions^13^ as well as with finite-size, composable security against coherent attacks under the same assumption of a memoryless untrusted device^14^.

So far experimental continuous-variable implementations were only guaranteed to be secure against so-called ‘collective attacks'^15^,^16^,^17^,^18^. While this class of attacks already allows an eavesdropper to possess a quantum memory, all quantum states are attacked identically using a collective Gaussian operation. Although Gaussian collective attacks are in the limit of an infinite number of distributed quantum states as strong as coherent attacks, it is currently not known whether this holds for a realistic finite key length protocol. For collective attacks a transmission distance of 80 km was achieved with a finite number of distributed quantum states using Gaussian modulated coherent states^18^,^19^. Previous proofs did also find composable security against coherent attacks for CVs^20^,^21^ but only for an unrealistically large number of distributed quantum states.

Here we report a continuous-variable QKD implementation that generates a finite and composable key that is secure against coherent attacks and whose security is furthermore 1sDI under memoryless assumption. The security of our implemented protocol is based on an extension of the security proof in ref. 14 including measurement flaws in the trusted detector. Our implementation is based on Gaussian Einstein–Podolsky–Rosen (EPR) entangled light and homodyne detection as considered in the security proof. An optimized, highly efficient error reconciliation algorithm was developed to enable the generation of the secret key.

## Results

### Robustness against implementation side channels

The 1sDI QKD implementation presented here is very robust against implementation side-channel attacks. It is secure against memoryfree attacks performed on Bob's untrusted detector, that is, attacks that are independent on Bob's previous measurement outcomes. This includes recently proposed attacks on the intensity of the local oscillator^22^,^23^, calibration attacks of the shot-noise reference^24^,^25^, wavelength attacks on the homodyne beam splitter^26^,^27^ and saturation attacks on the homodyne detector's electronic circuit^28^. Furthermore it is secure against Trojan-horse attacks on the source that usually threaten electro-optical modulators commonly used in Gaussian-modulation QKD protocols^29^,^30^. Placing the EPR source at Alice's station and assuming that her station is private and inaccessible to the eavesdropper by other means than the quantum channel^6^, prevents exploiting side channels related to the local oscillator used by Alice's trusted detector as the eavesdropper simply has no way of accessing it. Saturation attacks on Alice's homodyne detector are directly prevented by the security proof that includes an upper and lower bound for measurement outcomes^14^,^28^.

### EPR source

**Figure 1:**
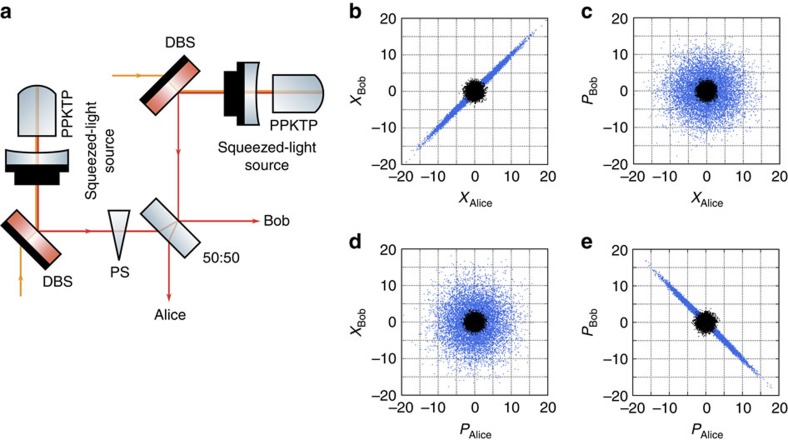
EPR entanglement source for CV QKD. (**a**) The source consists of two continuous-wave squeezed vacuum beams, generated by type I parametric down conversion at 1,550 nm (red), which are superimposed at a balanced beam splitter with a relative phase of 
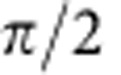
. Yellow beam: 775 nm pump field, DBS: dichroic beam splitter, PS: phase shifter. (**b**–**e**) Correlations between Alice's and Bob's data, measured by balanced homodyne detection in either the amplitude (*X*) or phase (*P*) quadrature. The data is normalized to the noise s.d. of a vacuum state. Blue: EPR entangled state used for QKD. Black: Reference measurement of zero-point fluctuations of the ground state (vacuum).

Our implemented protocol uses two continuous-wave optical light fields whose amplitude and phase quadrature amplitude modulations were mutually entangled^31^, produced by a source which is the only component in the set-up that is not compatible with existing telecommunication components. Using EPR entanglement as a resource makes our protocol a CV equivalent of the BBM92 protocol for discrete variables^32^. The schematic of the experimental set-up is illustrated in Fig. 1a. Two squeezed-light sources^33^,^34^, each composed of a nonlinear PPKTP crystal and a coupling mirror, were pumped with a bright pump field at 775 nm (yellow) to produce two squeezed vacuum states at the telecommunication wavelength of 1,550 nm (red). The two squeezed vacua, both exhibiting a high squeezing of more than 10 dB, were superimposed at a balanced beam splitter with a relative phase of *π*/2, thus generating EPR entanglement^31^. One of the output modes of the beam splitter was kept by Alice, while the other was sent to Bob. The technical details of the source, including the locking scheme, were characterized in ref. 35.

Figure 1b–e shows the distribution of measurement outcomes obtained by the two parties measuring either the amplitude (*X*) or phase (*P*) quadrature of their respective light field with balanced homodyne detection. Each measurement outcome is truly random since it stems from parametrically amplified zero-point fluctuations. When both parties simultaneously measure either *X* or *P* the strong correlations between their outcomes are clearly visible (Fig. 1b,e). If the two parties measure different quadratures instead, the measurement outcomes are uncorrelated (Fig. 1c,d). The strength of the correlations of Alice's and Bob's measurement for the same quadratures, which is related to the initial squeezing strength, is a central parameter in our QKD protocol and enters the key length computation directly in the form of an average distance *d*_pe_, introduced below.

**Figure 2:**
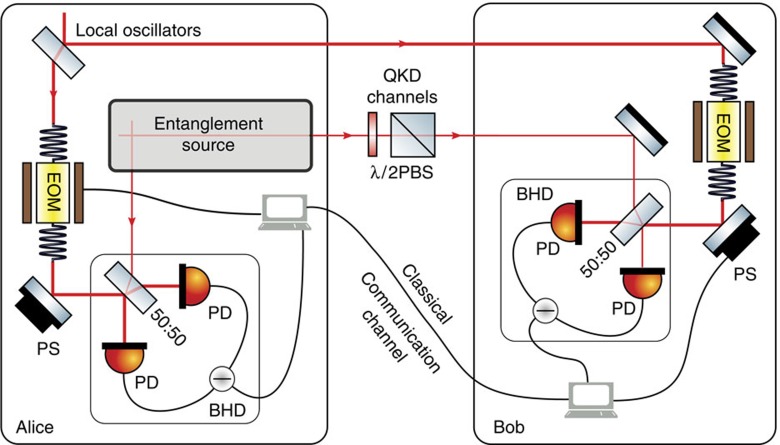
Implementation of Alice's and Bob's QKD receivers. Both parties used balanced homodyne detection (BHD) to measure their part of the quadrature entangled state. The measured quadrature angle was controlled by a computer via a fast fibre-coupled electro-optical modulator (EOM). To make sure that Alice and Bob switched between the same orthogonal quadratures, a phase shifter (PS) was employed to compensate slow phase drifts (see Methods section). Optical losses of the transmission channel to Bob were modelled by a variable attenuator consisting of a half-wave plate (*λ*/2) and a polarizing beam splitter (PBS). The measurement rate was 100 kHz. PD, photo diode.

A schematic of the experimental QKD set-up is shown in Fig. 2. The entanglement source was located at Alice's station and the local oscillators used for homodyne detection of the two entangled modes were generated locally at her station as well. While this assured that Alice's local oscillator was inaccessible to an eavesdropper, Bob's local oscillator was sent from Alice to Bob via a free-space channel. Both local oscillators had a power of 10 mW each. Implementation details can be found in the Methods section.

### Precise steps of the QKD protocol

*Preliminaries*. Alice and Bob use a pre-shared key to authenticate the classical communication channel for post-processing^36^. Furthermore, Alice and Bob negotiate all parameters needed during the protocol run and Alice performs a shot-noise calibration measurement by blocking the signal beam input of her homodyne detector.

*Measurement phase*. Alice prepares an entangled state using her EPR source and sends one of the output modes to Bob along with a local oscillator beam. Both Alice and Bob choose, randomly and independently from each other, a quadrature *X* or *P*, which they simultaneously measure by homodyne detection of their light fields. The outcome of this measurement is called a sample. This step is repeated until 2*N* samples have been obtained.

*Sifting*. Alice and Bob announce their measurement bases and discard all samples measured in different quadratures.

*Discretization*. The continuous spectrum of the measurement outcomes is discretized by the analogue-to-digital converter used to record the measurement. During the discretization step, Alice and Bob map the fine grained discretization of their remaining samples caused by the analogue-to-digital converter to a coarser one consisting of 2^*d*^ consecutive bins. In the interval [−*α*, *α*] a binning with equal length is used, which is complemented by two bins (−∞, −*α*) and (*α*, ∞). The parameter *α* is used to include the finite range of the homodyne detectors into the security proof.

*Channel parameter estimation*. The secret key length is calculated using the average distance between Alice's and Bob's samples. To estimate it, the two parties randomly choose a common subset of length *k* from the sifted and discretized data, 
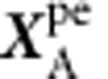
 and 
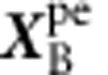
, respectively, which they communicate over the public classical channel. Using these, they calculate


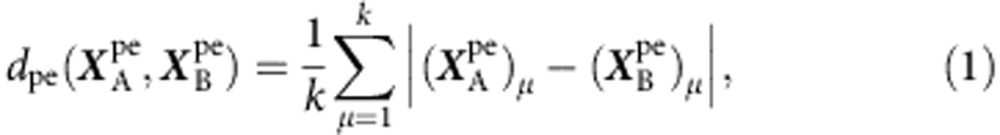


and abort if it exceeds a threshold agreed on in the preliminaries step.

*Error reconciliation*. Bob corrects the errors in his data to match Alice's using the hybrid error reconciliation algorithm described below. Later, Alice and Bob confirm that the reconciliation was successful.

*Calculation of secret key length*. Using the results from the channel parameter estimation and considering the number of published bits during error reconciliation, Alice and Bob calculate the secret key length 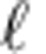 according to the presented secret key length formula in the Methods section. If the secret key length is negative, they abort the protocol.

*Privacy amplification*. Alice and Bob apply a hash function that is randomly chosen from a two universal family^37^, to their corrected strings to produce the secret key of length 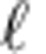.

### Assumptions of the security proof

The assumptions of the security proof on our implementation are the following: (1) Alice's station is a private space^6^ and Bob's station is isolated, that is, neither Bob's measurement choice nor his measurement results are leaking his station. (2) The energy of Alice's mode of the EPR state is bounded which allows Alice to determine the probability for measuring a quadrature amplitude value exceeding the parameter *α*. (3) Alice switches her homodyne detector randomly between two orthogonal quadratures (*X* and *P*) with 50% probability. (4) Bob is choosing randomly between two measurements that are assumed to be memoryless. (5) The phase noise present in Alice's measurement is Gaussian distributed with variances *V*_*X*_ and *V*_*P*_ for the amplitude and phase quadrature, respectively.

The first assumption is natural to (almost) all QKD implementations. The second one is assured in our implementation by placing the EPR source into Alice's station. For the third and fourth assumptions two independent quantum random number generators located at Alice's and Bob's stations were employed. For implementation details we refer to the Methods section. While Bob is choosing randomly between two measurements, it is not required that they are orthogonal quadrature measurements. Since the security of the key is independent of the actual measurements, an eavesdropper may temper with the local oscillator sent to Bob. In an experimental implementation phase noise is unavoidable, hence the security proof of ref. 14 has been extended, see Methods section for details. We characterized the phase noise in our implementation before the run of the protocol, showed that the quadratures are indeed Gaussian distributed and determined the variances to *V*_*X*_=*V*_*P*_≈(0.46°±0.01°)^2^. Details are given in the Methods section. Thus, our implementation fulfills all requirements of the security proof and the key generated by the above protocol is *ɛ*-secure against coherent attacks, where *ɛ* is the so-called composable security parameter.

### Error reconciliation protocol

Important for a high key rate is an error reconciliation protocol, which has an efficiency close to the Shannon limit. Since in our CV QKD protocol the discretized sample values are non-binary and follow a Gaussian distribution, error reconciliation codes with high efficiency and low error rate are more difficult to achieve than for discrete-variable protocols with uniformly distributed binary outcomes^17^. To solve the problem, we designed a two-phase error reconciliation protocol that can exploit the non-uniform distribution efficiently. First the *d*_1_ least significant bits of each sample are sent to Bob. Since these bits are only very weakly correlated, this step works with an efficiency very close to the Shannon limit. In a second step Alice and Bob use a non-binary low density parity check (LDPC) code over the Galois field 
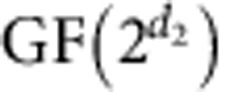
 to correct the *d*_2_=*d*−*d*_1_ most significant bits. *d*_1_, *d*_2_, as well as the LDPC code were optimized for the different channel conditions and the actually employed code was determined using the *k* revealed samples from the channel parameter estimation. More details are given in the Methods section.

### Secret key generation

**Figure 3:**
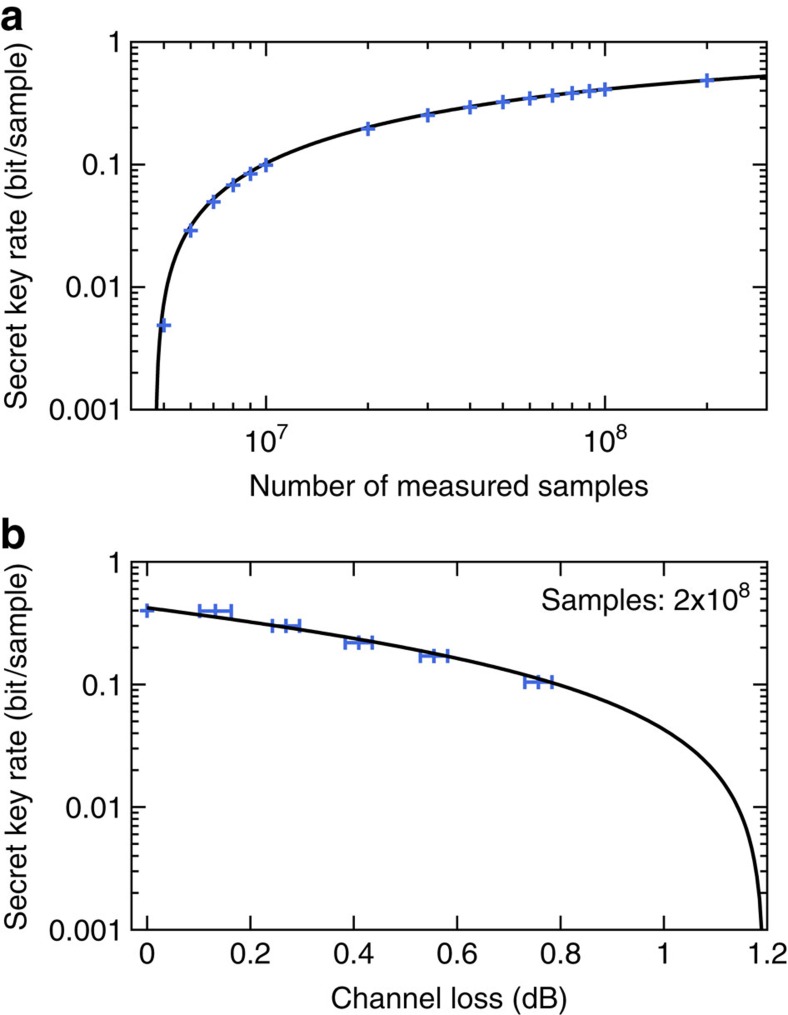
Secure key rates achieved by our CV QKD system. Common parameters: *α*=61.6, *d*=12, *ɛ*=2 × 10^−10^. (**a**) Effect of the finite number of distributed quantum states on the secret key rate. The graph shows experimental results (blue points) obtained without the variable attenuator in Bob's arm. The theoretical model (solid line) is included for comparison and was calculated by reconstructing the covariance matrix for 10^8^ samples. (**b**) Experimentally obtained secure key rate versus optical attenuation in the transmission line to Bob's detector for 2 × 10^8^ measured samples (blue points). The error bars (s.d.) are owing to the accuracy of the measurement of the optical attenuation. The theoretical model (solid line) was calculated by reconstructing the covariance matrix of the state corresponding to no attenuation (0 dB) and using a reconciliation efficiency of *β*=94.3%.

Figure 3 shows the experimental results. First we removed the variable attenuator in the transmission line to Bob and executed the protocol for different sample sizes to show the effect of the finite sample size on the secure key rate (Fig. 3a, blue points). For each sample size the number of samples *k* used for channel parameter estimation was optimized before each run of the QKD protocol to yield maximum key length. The hybrid error reconciliation had a total efficiency of *β*=94.6% without a single frame error. While we achieved a positive secret key rate with already 5 × 10^6^ samples, the secret key rate of 0.485 bit per sample achieved for 2 × 10^8^ samples is close to saturation. The theoretical model, which is the solid line in the figure, is shown for comparison.

With the variable attenuator in place, we varied the optical loss of the channel to Bob between 0 and 16% (Fig. 3b), which is equivalent to a fibre length of up to 2.7 km when standard telecommunication fibres with an attenuation of 0.2 dB km^−1^ are used and a coupling efficiency of 95% is taken into account. By measuring a total of 2 × 10^8^ samples we were still able to achieve a secret key rate of about 0.1 bit per sample at an equivalent fibre length of 2.7 km (≈0.76 dB channel loss). This value, as well as the secret key sizes at the other attenuation values, were achieved by having a very high overall error reconciliation efficiency between *β*=94.3 and 95.5%, again without a single frame error. The theoretical model shown in the figure reveals that even an optical transmission loss of almost 1.2 dB between Alice and Bob should be possible. This corresponds to an equivalent distance of about 4.8 km, which is already enough to implement CV QKD links with composable 1sDI security against coherent attacks between parties in, for instance, a city's central business district.

## Discussion

In conclusion, we have successfully implemented continuous-variable QKD with composable and 1sDI security against coherent attacks. Along with the exploitation of strong EPR entanglement and a new highly efficient error reconciliation algorithm, the innovation of fast controlled random switching between the two measured quadrature angles with low phase noise made the implementation possible. While in our set-up Alice and Bob were located on the same optical table, they could in principle be separated and connected by a standard telecommunication fibre (see Methods section).

Estimations show that our implementation is limited to about 4.8 km. Longer distances will be possible by using optical fibres with less loss, or by using reverse reconciliation where about 16 km are possible with a similar set-up^38^. Remaining secure against coherent attacks in the finite-size regime over even larger distances requires new security proofs since the uncertainty principle employed here yields a secret key rate that does not converge with number of distributed quantum states to the rate achieved for collective attacks and other currently available proofs require an unfeasibly large number of distributed quantum states. Even more impact will have a further developed proof that keeps all features demonstrated here, but avoids the requirement for an EPR source. It might be based on Gaussian modulation of coherent states^39^ instead, thus, making 1sDI QKD implemenations with composable security against the most general attacks possible that are solely based on telecommunication components.

## Methods

### Details of the experimental set-up

The measurement rate of our implementation was 100 kHz. For each measurement, both Alice and Bob had to choose randomly between the *X* and *P* quadrature. The necessary relative phase shifts of *π*/2 of the local oscillator with respect to the signal beam were applied to the local oscillator beam by a high-bandwidth fibre-coupled electro-optical phase modulator driven by a digital pattern generator PCI-Express card. Since not only the orthogonality of the measurements is important but also that Alice and Bob measure the same set of quadratures, we compensated slow phase drifts by a phase shifter made of a piezo attached mirror. The error signal for this locking loop was derived by employing an 82 MHz single sideband from the entanglement generation^35^ that was detected by the homodyne detector. By lowpass filtering the demodulated homodyne signal at 10 kHz with a sufficiently high order, the high frequency phase changes from the fibre-coupled phase modulator were averaged over. To make the average independent of the chosen sequence of quadratures we used the following scheme. For a choice of the *X* quadrature, the phase modulator was first set to a phase of *π*/2 during the first half of the 10 μs interval, and then to 0. For the *P* quadrature, the phase was first set to 0 and then to *π*/2. Thus, this scheme made sure that the phase did not stay in one quadrature for longer than 10 μs even in the case where one party chose by chance to measure only one quadrature for a while. The measurement was performed synchronously by Alice and Bob in the second half of the interval after 3 μs settling time.

The data acquisition was triggered by the pattern generator and performed by a two channel PCI-Express card at a rate of 256 MHz. The 200 acquired samples per channel were digitally mixed down at 8 MHz, lowpass filtered by a 200-tap finite impulse-response filter with a cutoff frequency of 200 kHz and downsampled to one sample. After the total number of samples were recorded the classical post-processing of the QKD protocol was performed.

Alice and Bob both employed a local oscillator with a power of 10 mW, yielding a dark noise clearance of about 18 dB. The efficiency of both homodyne detectors was 98% (quantum efficiency of the photo diodes 99%, homodyne visibility 99.5%). The pump powers for the two squeezed-light sources were 140 and 170 mW, respectively.

The optical attenuation of the variable attenuator used in Fig. 3b was measured by determining the strength of the 35.5 MHz phase modulation used to lock one of the squeezed-light sources^35^ with Bob's homodyne detector. The error bars in the figure are due to the accuracy of this measurement.

While in our implementation both parties were located on the same optical table and the quantum states including the local oscillator for Bob's homodyne detection were transmitted through free space, a separation is in principle possible by using standard telecommunication fibres. To send both the entangled state and the local oscillator to Bob, they could be, for instance, time multiplexed. Using a dedicated fibre for both beams would also be possible. To achieve synchronization between the two parties, a modulated 1,310 nm beam could be employed that could be sent along with the local oscillator by wavelength division multiplexing.

### Determination of Alice's homodyne measurement phase noise

**Figure 4:**
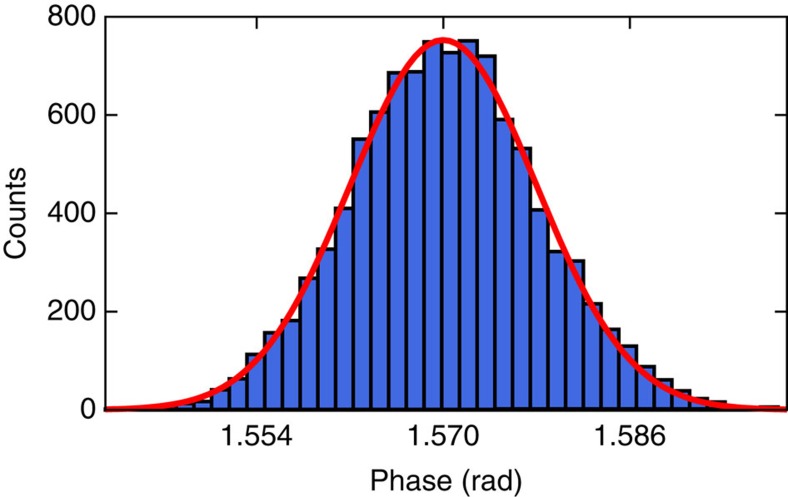
Phase noise measurement result. The s.d. of the fitted Gaussian function (red solid line) is 0.46°±0.01°.

The measurement of the phase noise of Alice's homodyne detection during random switching between the *X* and *P* quadrature was performed by measuring the beat between the local oscillator and the bright control beam that was used to lock the squeezed-light sources. Scanning the local oscillator's phase yielded a calibration between the measured output voltage of the homodyne detector's circuit and the phase angle between local oscillator and signal field. Measurements were taken with an oscilloscope while randomly switching the quadrature. As for the quadrature measurements (see above) a segment of 1 μs was taken 3 μs after switching quadratures and the mean value was calculated. Since the local oscillator was switched randomly between the *X* and *P* quadrature the phase noise is symmetric between the quadratures, hence *V*_*X*_=*V*_*P*_. Figure 4 shows a histogram of the phase noise measurement for 10^5^ samples. The red solid line shows a fit of a Gaussian distribution. The s.d. of the phase noise was determined to (0.46±0.01)°, which is quite low despite the randomly switched quadrature angle^34^. Thereby the error was determined by bootstrapping 1,000 data points from a total of 10,000.

### Quantum random number generator

The security of the protocol relies on the use of true random numbers that are needed by Alice and Bob to choose between the *X* and *P* quadrature, and to determine a random hash function during privacy amplification. We implemented a quantum random number generator following a scheme from ref. 40, which is based on vacuum state measurements performed by a balanced homodyne detector. For this purpose we implemented another balanced homodyne detector with blocked signal port using an independent 6 mW 1,550 nm beam from a fibre laser as local oscillator. The output of the homodyne detector circuit was anti-alias filtered by a 50 MHz fourth-order Butterworth filter and sampled with a sampling frequency of 256 MHz by a data acquisition card. The data was subsequently mixed down digitally at 8 MHz, lowpass filtered with a 200-tap finite-impulse-response filter with a cutoff frequency of 5 MHz and downsampled to 2 MHz. The generation of the random numbers from the data stream followed the procedure in ref. 40.

### Security proof considering measurement flaws

We use the security proof from ref. 14 and generalize it to phase errors in Alice's measurement of *X* and *P*. It has been shown that if the protocol passes, a secure key of length^14^


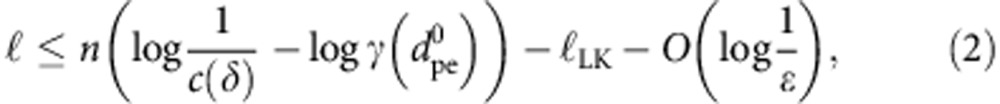


can be extracted. Here, *n*=*N*−*k* is the number of samples used for the key generation, *γ* is a bound on the correlation between Alice and Bob depending on the previously agreed average distance threshold 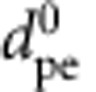 and 
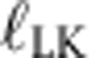
 is the number of communicated bits in the error correction protocol. The only term depending on Alice's measurement device is *c*(*δ*), which refers to the overlap of the discretized *X* and *P* measurements performed by Alice. In case of ideal *X* and *P* measurements satisfying the commutation relation [*X*, *P*]=*i*ℏ one obtains *c*(*δ*)≤*δ*^2^/(2*π*ℏ), where equality holds approximately for relevant sizes of δ.

Let us now assume that owing to experimental imperfections the actual measurements *X* and *P* deviate by a phase *θ*_*X*_ and *θ*_*P*_ from the ideal measurements, where *θ*_*X*_ and *θ*_*P*_ are distributed according to a Gaussian distribution with variance *V*_*X*_ and *V*_*P*_ centred at 0. Then we find that *X* and *P* satisfy the canonical commutation relation [*X*, *P*]=*i*ℏ′ with ℏ′=ℏcos*θ*, *θ*=*θ*_*X*_+*θ*_*P*_. This then results in an overlap *c*(*δ*, *θ*)=*δ*^2^/(2*π*ℏ′)=*c*(*δ*)/cos*θ*.

Considering *n* independent measurements, we obtain


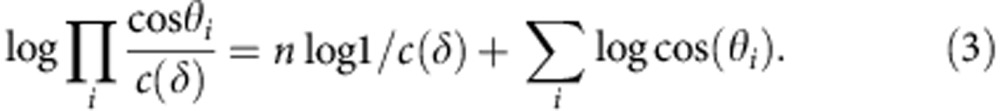


Using that log cos(*θ*)≥−*θ*^2^/(2ln2), we can bound 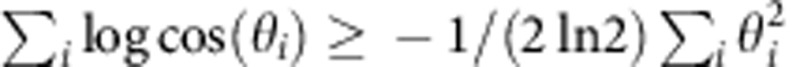 and Hoeffding's inequality yields that 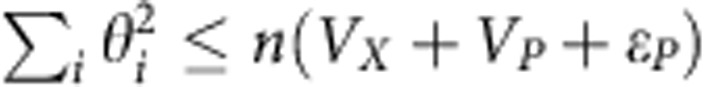 with probability exponentially small in 
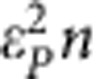
. Here we assumed that *θ*_*X*_ and *θ*_*P*_ are independent so that the expectation of *θ*^2^ is *V*_*X*_+*V*_*P*_. Plugging this into (2), we find that for Gaussian phase noise with variances *V*_*X*_ and *V*_*P*_ a secure key of length


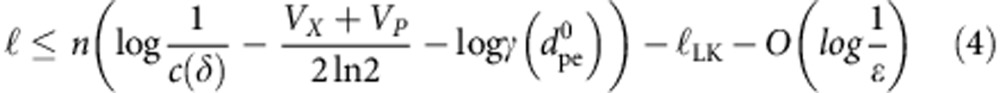


can be generated.

### Classical post-processing

The main post-processing is performed with the AIT QKD software. For the current protocol the following algorithms are combined: (i) the binning of the synchronized outcomes, (ii) the estimation algorithm for CV QKD, (iii) the reconciliation algorithm for CV QKD, (iv) the confirmation algorithm and (v) the privacy amplification algorithm. All classical messages during the protocol are authenticated with a message authentication code using a pre-shared secret key to select a random function from a set of (almost strongly two universal) polynomial hash functions.

(i) First, Bob's samples in the *P* quadrature are multiplied by −1 to account for the anti-correlation. Alice and Bob then discretize their sifted samples into 2^*d*^−2 bins of equal size *δ* in the interval [−*α*, *α*], and two additional bins (−∞, −*α*) and (*α*, ∞). The 2^*d*^ bins are identified with the key generation alphabet *χ*_kg_={0, 1}^*d*^ and each bin (symbol) has a unique binary representation of *d* bits. Alice and Bob obtain the binned sifted samples 
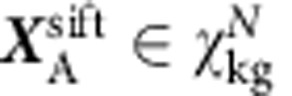
 and 
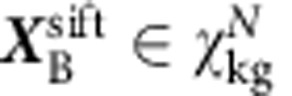
, respectively. Throughout the experiment we have used a key generation alphabet of size 
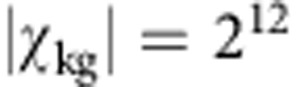
.

(ii) In the estimation module for CV QKD the average distance between Alice's and Bob's binned symbols is estimated. Alice chooses a random index set 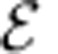
∑{1, 2,…, *N*} of size 
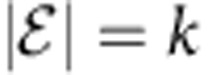
 for estimation and communicates 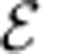 together with the corresponding binned symbols 
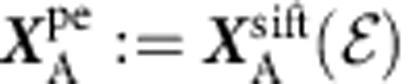
 to Bob. Bob determines his corresponding binned raw key symbols 
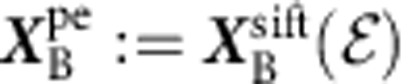
, calculates the mean difference *d*_pe_ between 
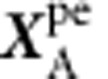
 and 
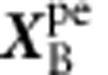
 (see equation (1)), and checks that 
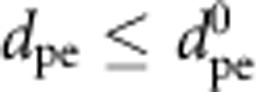
. Here, 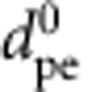 has been determined before the run of the protocol by a theoretical estimation given the characterization of the source, the fibre loss and excess noise. If the test passes they continue with the protocol and both parties remove the *k* estimation samples from their sifted samples to form their raw keys 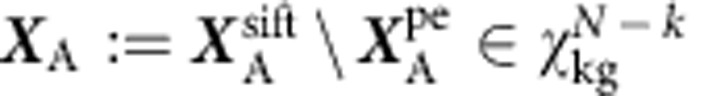 and 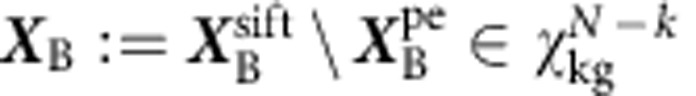.

(iii) The reconciliation module for CV QKD implements the hybrid reconciliation protocol. As the security analysis uses direct reconciliation, Bob has to correct his raw key ***X***_B_ to match with Alice's ***X***_A_ to generate a common raw key ***X***. The hybrid reconciliation used to correct Bob's noisy raw key operates directly on the key generation alphabet *χ*_kg_. In preparation for the hybrid reconciliation, two additional alphabets 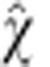 and 
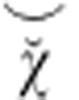
 are introduced such that 
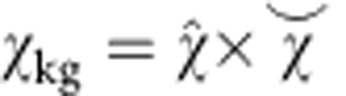
. Hence, each symbol *x*∈*χ*_kg_ has a unique decomposition 
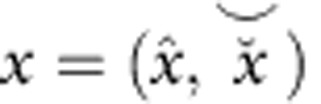
 with 
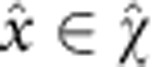
 and 
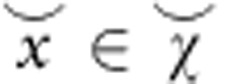
. We take for 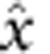 the *d*_2_ most significant bits of the binary representation of *x*, and for 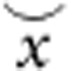 the remaining *d*_1_=*d*−*d*_2_ least significant bits of the binary representation of *x*. We thus decompose the raw keys as 
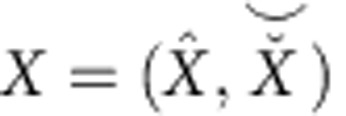
, where 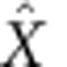 and 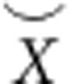 denote the sequence of the *d*_2_ most and the *d*_1_ least significant bits of each key symbol, respectively. The reconciliation module performs the following steps:

(iiia) On the basis of the variance of her binned raw key and the samples 
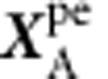
 and 
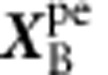
, Alice determines *d*_1_, *d*_2_, and the code rate *R* such that the expected leakage is minimized with respect to the entropy in Bob's symbols, and transmits these parameters to Bob.

(iiib) Then Alice communicates 
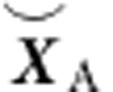
 to Bob who reconciles 
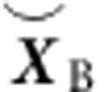
 simply by setting 
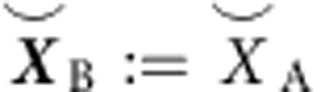
. Hence, the errors that are left in Bob's key ***X***_B_ are reduced to the errors in 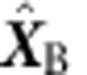. Non-binary LDPC reconciliation is used to correct 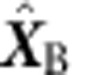 as described in the next step.

(iiic) Both Alice and Bob split their 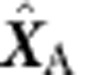 and 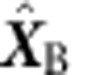 into blocks 
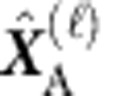
 and 
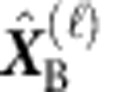
, 
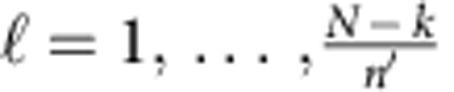
, each with *n*′=10^5^ elements of 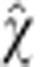. For this step we identify 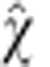 with GF
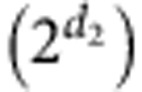
, the Galois field with 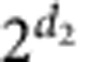 elements. For each block 
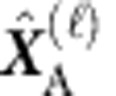
, Alice uses the parity check matrix *H̃* of an LDPC code over GF
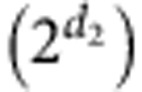
 and rate *R* to calculate the syndrome 
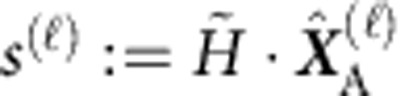
. Alice sends the syndrome 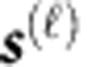 to Bob. For all elements 
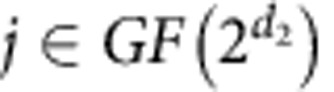
 and for all indices *i*∈{1,…,*n*} in the block Bob calculates the conditional probability that 
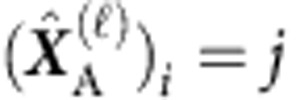
, given that Bob has obtained 
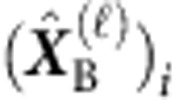
 and given Alice's value 
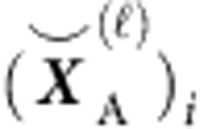
. Bob uses these probabilities to initialize a non-binary belief propagation decoder.

The non-binary belief propagation decoder operates in the probability domain using the multi-dimensional Hadamard transform to speed up the check node operations^41^. Using the syndrome 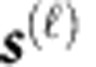 and the conditional probabilities mentioned above, this decoder calculates Bob's estimate 
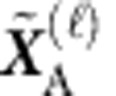
 of Alice's block 
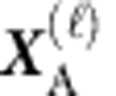
.

We have constructed parity check matrices of non-binary LDPC codes over Galois fields of order 32, 64, 128 and 256 with code rates *R*∈{0.50, 0.51,…, 0.95}. Each LDPC code has a variable-node degree of two, is check concentrated, and has a block length of 10^5^ symbols. We used the progressive edge-growth algorithm^42^ to construct binary codes in a first step. Then each edge has been assigned a random non-zero element of the corresponding Galois field^42^. Alice and Bob have access to all non-binary parity check matrices.

In our proof-of-principle experiment the error reconciliation step took about 2 h on a single central processing unit (CPU) core for the largest data set of 2 × 10^8^ samples. Taking into account the about 30 min to measure the data, real-time error reconciliation could in principle be achieved by splitting the task to, for example, five CPU cores. Alternatively, to speed up the computation an LDPC decoder algorithm with reduced complexity could be employed^43^.

(iv) After each block has been corrected, a confirmation step establishes the correctness of the protocol using a family *H* of (almost) two universal hash functions with 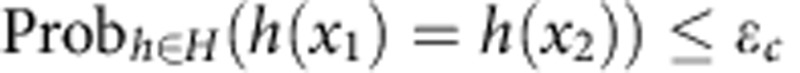 for all *x*_1_≠*x*_2_. For each block Alice chooses a hash function *h* randomly from *H* and communicates her choice to Bob. Alice and Bob apply this hash function to their blocks 
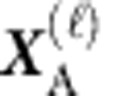
 and 
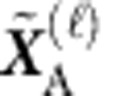
 and exchange the results. If their results agree the probability that Alice's and Bob's blocks are different is bounded from above by *ɛ*_*c*_. If their results disagree then their blocks are definitely different, and they discard them.

(v) Finally, Alice and Bob feed the sequence of all confirmed blocks into the privacy amplification module. Given the accumulated leakage 
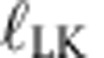
 in bits from the previous protocol steps, the secure key length 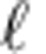 is calculated according to equation (4). Alice chooses a hash function randomly from a two universal hash family and communicates her choice to Bob. Then Alice and Bob both apply this hash function to the reconciled blocks and obtain the *ɛ*-secure key *K*_sec_.

## Additional information

**How to cite this article:** Gehring, T. *et al.* Implementation of continuous-variable quantum key distribution with composable and one-sided-device-independent security against coherent attacks. *Nat. Commun.* 6:8795 doi: 10.1038/ncomms9795 (2015).
